# Experimental Investigation on the Bonding Performance of Steel Bars in Desert Sand Concrete After Freeze–Thaw Cycles

**DOI:** 10.3390/ma18173971

**Published:** 2025-08-25

**Authors:** Min Li, Zhiqiang Li, Jian Jiao

**Affiliations:** 1 College of Water Conservancy & Architectural Engineering, Shihezi University, Shihezi 832003, China; minli1078@163.com; 2 College of Civil Engineering, Kashi University, Kashi 844000, China; jiaojianksu@163.com; 3 Xinjiang Key Laboratory of Engineering Materials and Structural Safety, Kashi University, Kashi 844000, China

**Keywords:** desert sand concrete, freeze–thaw cycles, pull-out test, bond performance, bond–slip constitutive model

## Abstract

Desert sand (DS) serves as a sustainable alternative to river sand in concrete production, delivering environmental and economic benefits. Furthermore, the durability of concrete structures in cold regions is severely affected by freeze–thaw (F-T) cycles. Therefore, this study employed a central pull-out test to examine the bond performance between desert sand concrete (DSC) and steel bars subjected to F-T cycles, considering the effects of the number of F-T cycles, DS replacement ratios (i.e., the replacement ratio of river sand by DS), and the type of reinforcement. The F-T cycle numbers tested were 0, 25, 50, and 75 cycles. The DS replacement ratios were varied at 0%, 20%, 40%, 60%, 80%, and 100%. The plain and threaded steel bars (PSBs and TSBs) were selected for the experiment. The results indicate a decrease in bond strength for both PSB and TSB specimens with increasing F-T cycle numbers. Regarding the DS replacement ratios, bond strength initially decreased, with an increasing replacement rate, then increased, and eventually reduced again. Notably, significantly improved bonding was observed for steel reinforcement in DSC containing 40% or 60% DS compared to plain concrete. Additionally, the bond strengths of PSB specimens were lower than those of TSB specimens under identical conditions. A calculation formula for the bond–slip characteristic was derived using statistical regression, which considered multiple factors. Eventually, a bond–slip constitutive model was developed for the interface between DSC and reinforced steel, showing a high degree of consistency with the experimental data.

## 1. Introduction

In cold regions, concrete structures have long faced severe freeze–thaw(F-T) cycles. F-T action is a primary contributor to concrete durability deterioration; the resultant internal damage significantly impairs the bond between concrete and reinforcing steel [1,2]. At the same time, with the large-scale development of global infrastructure, river sand resources are becoming increasingly scarce. Replacing natural river sand with Earth’s abundant desert sand (approximately 6 million square kilometers) in concrete has alleviated the shortage of river sand and combated the expansion of desertification [3,4,5]. However, compared to river sand, DS has a smaller particle size and a smoother surface. Due to the selective separation of wind, the particle size distribution of DS is poor, mainly composed of ultrafine and uniform particles [6,7]. Therefore, conducting experimental investigations on the bonding performance of steel bars in desert sand concrete (DSC) after F-T cycles is crucial for evaluating and improving the long-term service safety and durability of DSC structures in cold regions.

In recent years, a considerable number of researchers have achieved remarkable breakthroughs in the study of DSC. Li et al. [8,9,10,11] conducted experimental investigations on the bending and shear resistance of DSC beams, as well as the seismic behavior of frame joints and columns. Qin et al. [12] explored the effects of steel fiber volume content, DS replacement ratio, and reinforcement ratio on the flexural performance of DSC beams. Bekey et al. [13] studied the axial compression behavior of DS-filled steel-tube concrete columns. Wang et al. [14] demonstrated through experimentation that engineering cementitious composites (DS-ECC), prepared by fully replacing silica sand with DS and incorporating PVA fibers, significantly enhanced tensile ductility while maintaining mechanical properties comparable to those of traditional ECC. Li and Shen [15] and Dong et al. [16,17] examined how aeolian sand concrete durability degrades when exposed to complex environmental conditions. These studies collectively indicate that the load-bearing performance of DSC components is comparable to that of conventional concrete. Furthermore, the use of an appropriate amount of DS can lead to optimization of the pore structure and improvements in the durability of concrete. El Hasan et al. [18] found that DS completely replaces fine aggregates in 3D-printed concrete, with a compressive strength exceeding 40 MPa. Meng et al. [19] found that ECC containing DS exhibits tensile strain hardening behavior. In addition, Sahmaran et al. [20] found that ECC with DS100% replacement of ordinary sand had similar tensile strength at 14 days.

Reliable bonding performance between steel bars and DSC is essential to ensure composite action, and is a key factor in the structural application of DSC. Zhong et al. [21] studied how bond strength deteriorates at the DSC–steel reinforcement interface. Qin et al. [22] examined the influence of fly ash content and R on the bonding performance of reinforced concrete. These studies indicate that the content of DS significantly affects the bonding performance of DSC.

F-T cycles are a major factor contributing to the deterioration of concrete durability in cold regions [23]. During these cycles, water in the pores repeatedly freezes and thaws, causing internal microstructural damage and further compromising the integrity of concrete [24,25,26]. Consequently, examining the interfacial bonding characteristics between steel bars and DSC under F-T conditions is of critical importance. In recent years, many researchers have extensively investigated the bonding behavior at the concrete–rebar interface under F-T cycles. Hanjari et al. [27] found, using pull-out tests, that the bonding performance of frost-damaged concrete undergoes significant changes; that is, the slip at the maximum bonding stress increases with frost damage. Petersen et al. [28] conducted multiple tensile tests on reinforced-concrete specimens subjected to F-T cycles, and, based on the experimental results, adjusted and developed a local tensile load slip model for concrete and steel bars under F-T conditions. Alves et al. [29] tested the individual and combined effects of F-T cycling and sustained and fatigue loading conditions on the bond–slip behavior between GFRP steel bars and concrete. According to their results, F-T cycling increased adhesive strength by about 40%. Belarbi et al. [30] studied the comprehensive effects of F-T cycling, high-temperature cycling, and deicing salt solution on bond strength, with a focus on GFRP and CFRP steel bars in various types of concrete. They observed that the peak bond stress of GFRP steel bars decreased more significantly compared to CFRP steel bars. Khanfour’s study [31] showed that the bond strength between BFRP steel bars and concrete did not change significantly after 200 F-T cycles, demonstrating good bonding performance. Li et al. [32] examined how F-T cycling and recycled concrete content affect the peak bond strength and corresponding slip value at the recycled concrete–rebar interface. Wang et al. [33] examined how aggregate type affects BFRP–concrete bonding behavior using central pull-out tests in F-T environments. Li et al. [34] examined the bonding behavior of steel–FRP composite bars to marine sand concrete under varying cover thicknesses and F-T cycles, utilizing eccentric pull-out tests and developing an associated bond–slip constitutive model. Huang et al. [35] experimentally and theoretically investigated the durability of recycled aggregate concrete (RAC) under F-T conditions in cold marine environments, along with the bond degradation of corroded steel reinforcements in RAC, and proposed a predictive bond–slip model. Liu et al. [1] examined the influence of F-T cycles and concrete compressive strength on the bonding characteristics of reinforcement in concrete laterally restrained by stirrups, and established both a constitutive bond–slip model and a formula for calculating bond strength.

Currently, while extensive research has explored the post-F-T bond between reinforced steel and conventional concrete, studies on the DSC reinforcement bond under comparable conditions remain relatively scarce. To address this gap, the present study designed 48 groups, comprising 144 central pull-out specimens, to examine the effects of F-T cycles, DS replacement ratios, and steel bar type on bond anchorage performance. Furthermore, a bond–slip constitutive model was established to provide a theoretical basis and technical reference for the broader application and promotion of DSC in structural engineering.

## 2. Experimental Overview

### 2.1. Specimen Design

**Figure 1:**
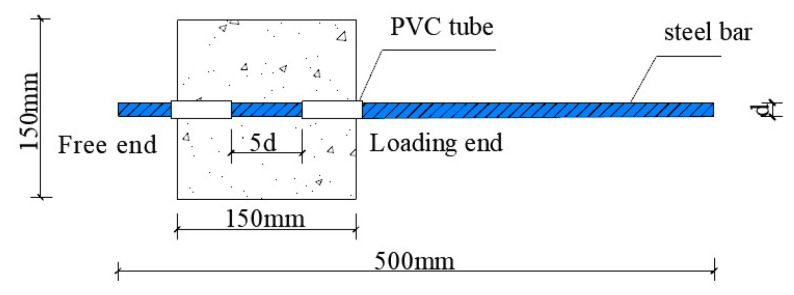
Geometric dimensions of the pull-out specimen.

In total, 48 sets of samples were designed (see Table 1), with three specimens in each set. A total of 144 specimens were designed for examining the post-F-T bar bonding behavior in DSC, considering the influence of F-T cycle number (N), DS replacement ratios (i.e., the replacement ratio of river sand by DS) (R), and reinforcement type. The F-T cycles tested included four values: 0, 25, 50, and 75 cycles. The DS replacement ratios were varied at six levels: 0%, 20%, 40%, 60%, 80%, and 100%. Two types of reinforcement (plain steel bar, PSB; threaded steel bar, TSB) were used for this experiment. For each specimen type, three replicates were used, resulting in a total of 144 specimens. The naming convention for each group of pull-out specimens was reinforcement type-R-N. For instance, PSB-R40-25 represents a plain steel bar specimen, where river sand is replaced by DS at 40% and exposed to 25 F-T (F-T) cycles. The geometric dimensions of all pull-out specimens were identical, as depicted in Figure 1. The concrete block was a 150 mm cube. The anchorage length (L) of the steel bar was 5d, where d was the steel bar diameter. To prevent local stress concentration at the loading end, PVC sleeves were placed on the non-bonded section of the steel bars.

### 2.2. Materials

**Figure 2:**
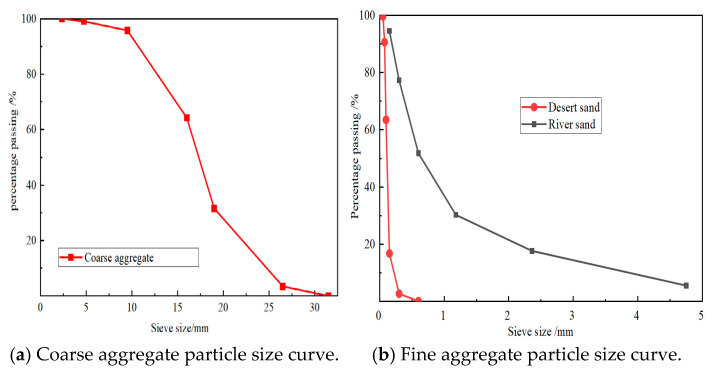
Sieve curve.

The concrete mix ratio is shown in Table 2. The coarse aggregate consisted of 5–25 mm crushed stone obtained from a screened and washed quarry in Shihezi, Xinjiang, China. Its physical properties are summarized in Table 3. The particle size distribution of coarse and fine aggregates is shown in Figure 2. The fine aggregate consisted of river and DS, and its physical properties are shown in Table 3. The P·O42.5R-grade cement and HSC polycarboxylic acid high-performance water-reducer were used. According to standard [36], the WAW-2000 kN electro-hydraulic servo universal testing machine produced by Tianshui Hongshan Testing Machine Co., Ltd., Tianshui, China, was used to test the compressive strength of concrete under different DS replacement ratios, as shown in Table 2. The grades of PSB and TSB used in the experiment were HPB400 and HRB400, respectively, with both bars having a diameter of 12 mm. According to standard [37], the mechanical properties of these steel bars were tested using the WAW-300 kN electro-hydraulic servo universal testing machine produced by Tianshui Hongshan Testing Machine Co., Ltd. (Table 4).

### 2.3. Test Setup and Method

**Figure 3:**
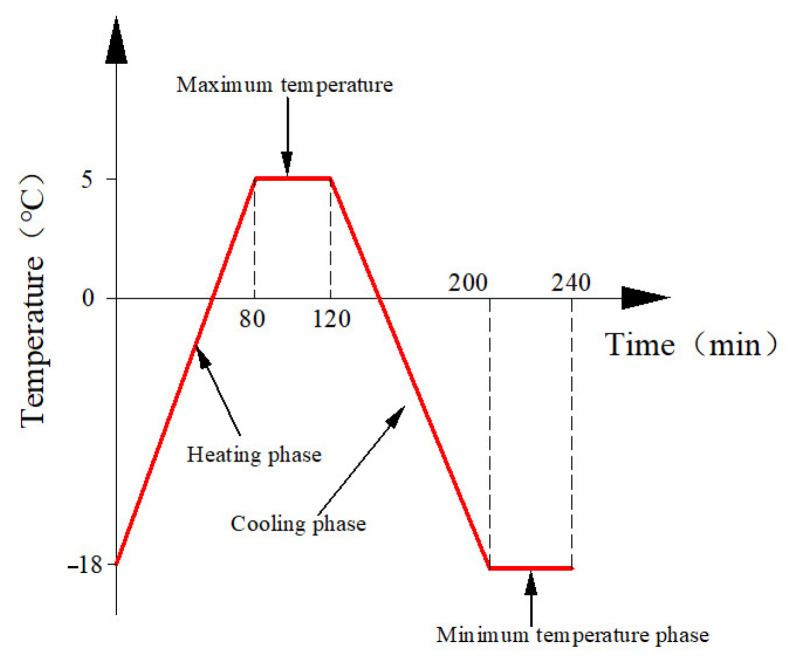
Temperature variation during a single freeze–thaw cycle.

**Figure 4:**
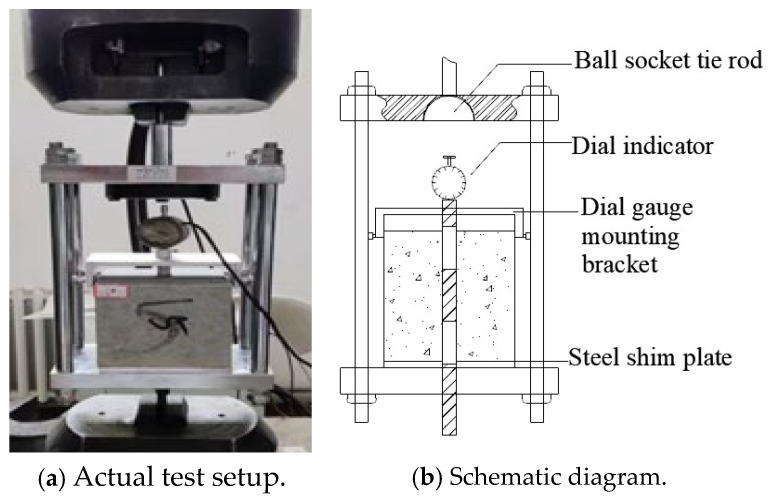
Test setup.

Before F-T cycling, all specimens underwent a two-stage conditioning process: initial curing, where, after 24 h of demolding, they were stored in a standard curing chamber (20 ± 2 °C) for 27 days to reach the 28-day maturation period, followed by water saturation treatment, immersing the specimens in water at 20 ± 2 °C for 4 days to ensure complete saturation. Subsequently, according to the Chinese national standard GB/T 50082-2024 [38], the rapid freezing method was used to conduct F-T cycles using the Tianjin Huida TDRF-1 concrete rapid F-T testing machine. Each F-T cycle lasted 240 min, comprising a 120 min freezing phase and a 120 min thawing phase. The minimum and maximum temperatures were set to −18 °C and 5 °C, respectively. During the thawing phase, specimens were heated from −18 °C to 5 °C, followed by a 40 min holding period at 5 °C. In the freezing phase, specimens were cooled from 5 °C to −18 °C, with a subsequent 40 min holding period at −18 °C. Figure 3 illustrates the temperature–time profile of a complete cycle. After completing the specified number of F-T cycles, the DDL600 electronic universal testing machine (maximum load 600 kN) from Changchun Institute of Mechanical Science was used for center pull-out testing. The test apparatus is shown in Figure 4. During loading, a displacement-controlled method was employed with a rate of 0.2 mm/min. A Dial Indicator displacement sensor was placed at the free end of the specimen to measure slip, while the load was automatically recorded using the force sensor of the testing machine.

## 3. Experimental Results and Analyses

### 3.1. Failure Mode

**Figure 5:**
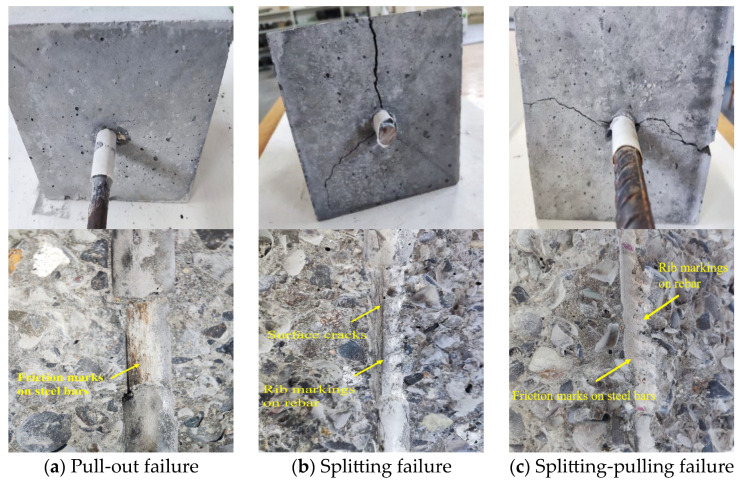
Failure modes.

Three failure modes were identified in the experiments, pull-out failure (P), splitting failure (S), and splitting–pulling failure (S + P), as displayed in Figure 5. The failure mode of the PSB specimens was pull-out failure. During loading, these specimens produced no significant noise, and no visible cracks were observed on the surface. However, a significant slip was detected at the free end. After cutting the concrete block, clear friction traces of the steel bars were visible. The failure mode of most TSB specimens was splitting failure. For specimens exhibiting splitting failure, no noticeable changes were observed on the surface at the initial loading stage. As the load increased, cracks gradually propagated from the loading end to the free end. The bearing capacity decreased rapidly upon reaching the ultimate load, accompanied by noticeable slippage and a distinct cracking sound. Additionally, some cracks that did not extend to the outer surface were observed on the bonding interface. Notably, some TSB specimens (e.g., TSB-R100-50 and TSB-R100-75) experienced splitting–pulling failure. The characteristics of splitting–pulling failure were generally similar to those of splitting failure. However, the crack widths corresponding to splitting–pulling failure at both the loading and free ends were smaller. This difference can be attributed to the excessive addition of DS, which weakened the mechanical interlock between the steel bars and the concrete. Furthermore, rising F-T cycles resulted in a progressively greater deterioration in concrete. In these cases, no clear sound was produced during failure, and friction traces on the steel bar ribs were evident on the bonding surface.

### 3.2. Ultimate Bond Strength

The average ultimate bond strength for each group of pull-out specimens is summarized in Table 5. Equation (1) was employed to calculate the ultimate bond strength for each specimen, with Fu denoting the peak load on the bond–slip curve.

(1) $$\tau_{u}=\frac{F_{u}}{\pi dl}$$

#### 3.2.1. Effect of Steel Bar Shape

**Figure 6:**
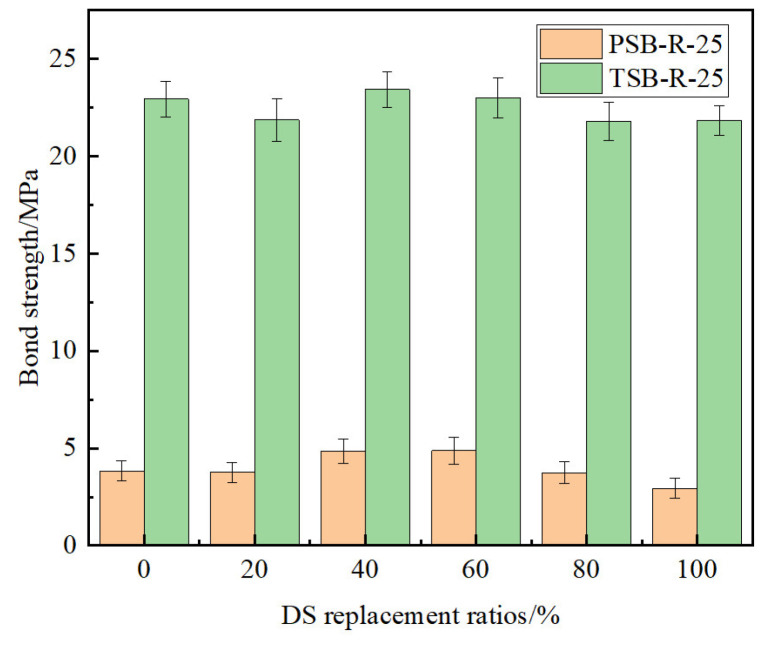
Effect of steel bar shape on ultimate bond strength.

As shown in Table 5, all PSB specimens exhibited pull-out failure, while the TSB specimens displayed two failure modes: splitting and splitting–pulling failure. Given equivalent DS replacement ratios and numbers of F-T cycles, TSB specimens exhibited markedly greater peak loads and ultimate bond strengths compared to their PSB counterparts. At DS replacement ratios of 0%, 20%, 40%, 60%, 80%, and 100%, bond strengths in TSB specimens following 25 F-T cycles showed increases of 498.96%, 482.93%, 384.09%, 371.72%, 484.45%, and 642.85%, respectively, relative to PSB specimens (Figure 6). This was attributed to the different bonding mechanisms between steel bars of varying shapes and DSC. The bonding between the PSB and DSC was primarily based on frictional and chemical bonding. In contrast, the bonding between the TSB with longitudinal and transverse ribs and DSC mainly consisted of frictional, chemical, and mechanical bonding. Mechanical bonding contributed to significantly higher bond strength in TSB specimens compared to PSB specimens [39].

**Figure 7:**
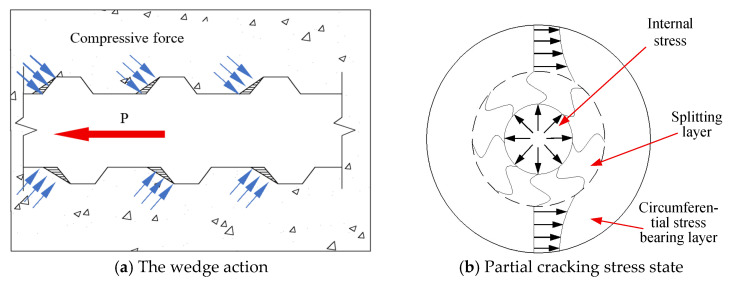
Schematic diagram of wedge action and covering layer spalling.

The mechanical bond of TBS specimens is provided by the diagonal compressive force exerted on the concrete by the transverse ribs. The force applied by the transverse ribs to the concrete forms a wedge shape, generating circumferential tensile stress in the surrounding concrete, ultimately leading to brittle longitudinal splitting of the specimen [40], as shown in Figure 7. Therefore, unlike PSB specimens, the vast majority of TSB specimens exhibit a splitting failure mode.

#### 3.2.2. Effect of DS Replacement Ratio

**Figure 8:**
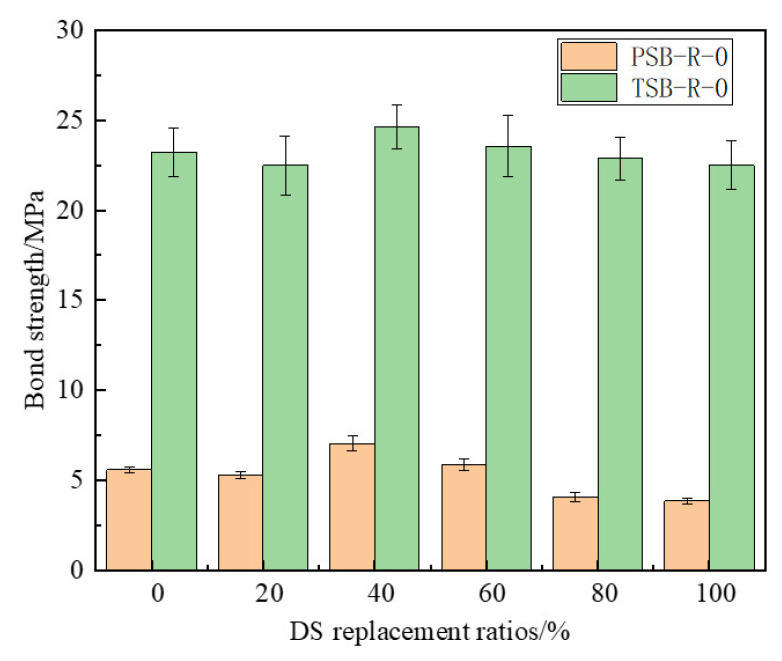
Effect of DS replacement ratio on ultimate bond strength.

DS replacement ratio significantly influenced bonding performance in the pull-out specimens. As shown in Figure 8, for specimens with the same reinforcement type, the bonding strength first decreased, then increased, and finally decreased again as the DS replacement ratio increased. Note that Figure 8 displays specimens without F-T exposure, illustrating the impact of DS replacement ratio on bond strength. At a 0% DS replacement ratio, PSB and TSB specimens exhibited ultimate bonding strengths of 5.6 MPa and 23.24 MPa, respectively. At a DS replacement ratio of 20%, PSB and TSB specimens showed bond strengths 5.18% and 3.22% lower, respectively, relative to specimens without desert sand replacement. This reduction was due to the smaller particle size and lower internal friction angle of desert sand. The lower DS replacement ratio weakened the mechanical bond between coarse and fine aggregates, thereby reducing mortar strength. However, bonding strength commenced an upward trend as DS replacement ratio reached 40%. At a 60% DS replacement ratio, the bonding strengths of the PSB and TSB specimens were 4.82% and 1.42% higher than those of specimens without desert sand replacement, respectively. This improvement was due to the high water absorption capacity of desert sand, which reduced the water–cement ratio of the concrete. Additionally, desert sand filled the pores within the concrete, making it more compact and thus increasing the bond strength. At an 80% DS replacement ratio, bond strength commenced a decline. At 100% desert sand replacement, PSB and TSB specimens exhibited bonding strengths reduced by 31.25% and 3.18%, respectively, relative to specimens without desert sand replacement. This decrease was attributed to the relatively low strength of desert sand particles. A high proportion of desert sand reduced the mortar strength, thereby weakening the bond between the mortar and the aggregates [41].

#### 3.2.3. Effect of Freeze–Thaw Cycles

**Figure 9:**
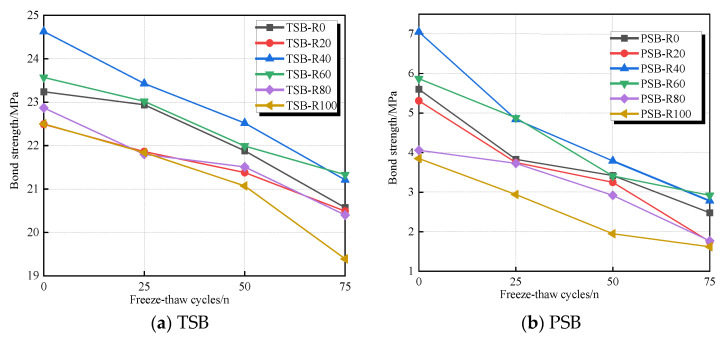
Effect of freeze–thaw cycles on the ultimate bond strength of specimens with different steel bar shapes.

The number of F-T cycles exerted a pronounced effect on the bond strength, which was observed to decline with increasing F-T cycles in Table 5 and Figure 9. Evidently, PSB specimens exhibited a more significant reduction in bond strength compared to their TSB counterparts. For concrete incorporating 0%, 20%, 40%, 60%, 80%, and 100% desert sand, the bond strength reductions in TSB specimens after 25 F-T cycles were 1.29%, 2.8%, 4.87%, 2.33%, 6.48%, and 2.93%, respectively; in comparison, reductions in bond strength for PSB specimens were 31.61%, 29.38%, 31.35%, 16.87%, 8.13%, and 23.64%, respectively. For concrete incorporating 0%, 20%, 40%, 60%, 80%, and 100% desert sand, bond strength reductions in TSB specimens after 75 F-T cycles were 11.49%, 8.89%, 13.89%, 9.5%, 12.48%, and 13.82%, respectively; however, PSB specimens suffered reductions of 50.71%, 67.04%, 60.57%, 50.26%, 56.40%, and 57.92%, respectively. These results can be explained as follows: At low temperatures, the free water in the concrete pores froze and expanded, generating internal stress that caused cracks in the transition layer between the concrete and steel bars to expand. As the temperature rose, the frozen water melted, creating osmotic pressure in the bonding layer. With repeated F-T cycles, microcracks inside the concrete continued to expand, reducing the frictional and mechanical bonding, consequently decreasing bond strength. With repeated F-T cycles, microcracks within the DSC continue to propagate and coalesce, forming extended crack networks with increased width. These cracks preferentially develop along the weakest paths. Furthermore, F-T damage induces a drastic reduction in the effective tensile strength of the bulk DSC, particularly at the concrete–rebar interface. The resultant crack networks facilitate hoop tensile stresses generated by radial components, which readily initiate severe longitudinal splitting cracks, ultimately contributing to premature bond failure.

Additionally, the bond strength of steel bars in DSC with DS replacement ratios of 40% and 60% was noticeably higher than that in ordinary concrete. This was because the DSC with replacement ratios of 40% and 60% was denser than ordinary concrete, containing fewer pores and microcracks. As a result, the DSC exhibited better frost resistance than ordinary concrete [41].

### 3.3. Peak Slip

**Figure 10:**
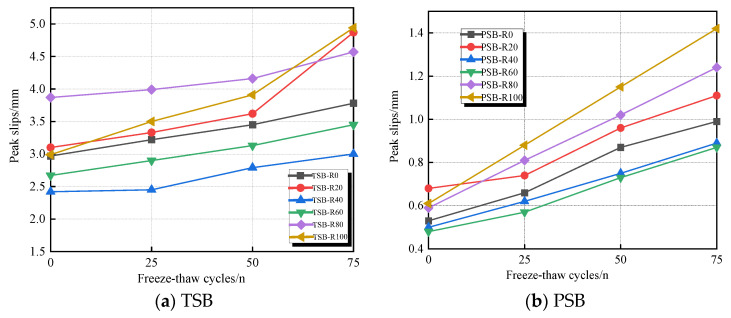
Peak slips of the pull-out specimens with different steel bar shapes.

The peak slip (*s_u_*) corresponds to the slip value at the ultimate bond strength. Table 5 and Figure 10 illustrate the average peak slip of all specimens. An increase in peak slip was observed when increasing the number of F-T cycles. For specimens undergoing the same number of F-T cycles, PSB specimens exhibited a higher rate of peak slip increase compared to TSB specimens. For concrete incorporating 0%, 20%, 40%, 60%, 80%, and 100% desert sand, increases in peak slip for TSB specimens after 25 F-T cycles were 8.42%, 7.42%, 1.22%, 8.61%, 3.10%, and 8.36%, respectively, while for PSB specimens, the increases were 24.53%, 8.82%, 24.00%, 18.75%, 37.29%, and 44.26%, respectively. For concrete incorporating 0%, 20%, 40%, 60%, 80%, and 100% desert sand, increases in peak slip for TSB specimens after 75 F-T cycles were 27.27%, 57.10%, 23.97%, 29.21%, 18.09%, and 65.22%, respectively; however, PSB specimens suffered increases of 86.79%, 63.24%, 78.00%, 81.25%, 110.17%, and 132.79%, respectively. Under the continuous F-T damage environment, the wedge effect produced by the combined action of concrete and the ribs of the rebars effectively restrained crack expansion. As a result, the degradation rate of bond behavior in the TSB specimens was slower than that in the PSB specimens. Additionally, the elastic modulus of DSC was lower than that of ordinary concrete [41], which led to an increased slip in the DSC specimens during the main crack formation process.

### 3.4. Bond Strength–Slip Curve

**Figure 11:**
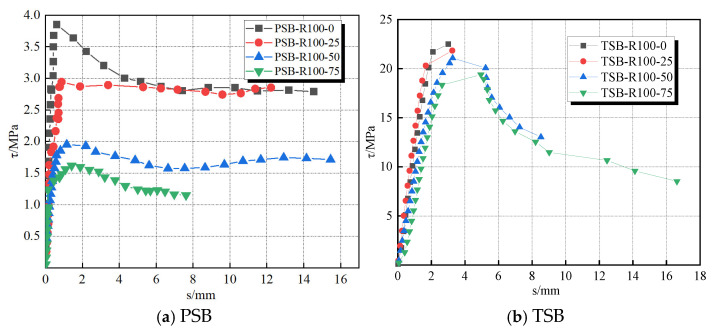
Bond strength–slip curves of the typical pull-out specimens with different steel bar shapes.

Figure 11a shows the bond strength–slip curves (i.e., *τ*-*s* curve) of several PSB specimens. These specimens manifest bond–slip curves progressing through three characteristic phases: the linear rising stage, the slip increase stage, and the debonding decline stage. In the initial loading stage (i.e., the linear rising stage), shear force began to develop at the bond interface between the steel bar and concrete. This stage witnessed a minor slip emergence at the specimen’s loaded end. Meanwhile, a linear relationship between shear force and slip was observed. With increasing load, the specimen advanced to the slip increase stage. In this stage, the chemical bonding between the steel bar and concrete gradually weakened. The debonding position extended progressively from the loaded end toward the free end. Additionally, the bond–slip curve displayed significant nonlinear characteristics. Once the applied load reached its peak, the specimen entered the debonding decline stage. At this point, the bond stress gradually decreased as slip increased. Notably, the chemical bonding had largely disappeared, and the bond force was primarily provided by frictional bonding.

**Figure 12:**
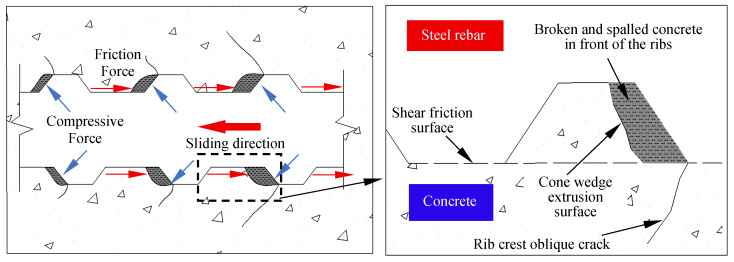
Schematic diagram of splitting–pulling failure mechanism.

The bond–slip curves of partially TSB specimens, shown in Figure 11b, exhibit similar characteristics to those of PSB specimens. Generally, the bond–slip curves of TSB specimens can be divided into three stages: the linear stage, the splitting failure stage, and the splitting–pulling stage. Notably, splitting failure specimens progressed through only the first two stages. In the initial loading stage (i.e., the linear stage), the loaded end slipped slightly, while the free end did not slip. This was because the bond force in this stage was provided by both chemical and mechanical bonding. Following F-T action, a significant reduction occurred in the chemical steel-concrete bonding, resulting in bond force dependence entirely on the mechanical bonding provided by the steel ribs. As the load increased, the specimen entered the splitting failure stage, which was marked by a decrease in the slope of the bond–slip curve. At this stage, the concrete in front of the inclined surface of the rib enters a plastic state, forming a wedge-shaped crushing zone. At the same time, due to the action of circumferential stress, longitudinal cracks will form at the weakest point of the concrete protective layer. When the applied load reaches its peak, the circumferential tensile stress generated by the radial component of the steel rib exceeds the tensile strength of the concrete, and internal microcracks penetrate to form longitudinal splitting cracks that extend along the axial direction of the steel bar to the surface. The vast majority of specimens rapidly undergo brittle failure and directly lose their load-bearing capacity. Only the remaining specimens TSB-R100-50 and TSB-R100-75 enter the splitting pulling stage. At this stage, there is a significant decrease in the load slip curves of the two specimens. This is because the specimens with a high DS replacement ratio have lower tensile strengths, and, after multiple rounds of F-T cycles, there are more microcracks inside the concrete, causing severe damage. The circumferential tensile stress is less than the tensile strength of the concrete. At this time, the concrete in front of the ribs is completely crushed into powder, forming a continuous slip channel. The concrete ridges between the ribs are cut off, and the steel bars are pulled out, as shown in Figure 12.

## 4. Bond–Slip Constitutive Model

The bond–slip constitutive relationship serves as a critical theoretical foundation for quantitatively characterizing the mechanical behavior at the steel–concrete interface and revealing their synergistic mechanism, forming the core of precise structural performance analysis. Based on systematic experimentation and regression analysis, this study incorporates the degradation effects of both the number of F-T cycles (N) and the DS replacement ratios (R) on interfacial bond strength. A constitutive model fully characterizing the entire interfacial slip process is established, providing a theoretical basis for predicting bond behavior between desert sand concrete and steel reinforcement under F-T cycles.

### 4.1. Statistical Regression of Bond–Slip Characteristic Values

As described in Section 3.4, the bond–slip curve of the pull-out specimen was divided into three stages. Thus, three characteristic points were defined: the initial slip point, the peak slip point, and the debonding point. The strengths at the initial slip and debonding points are represented by *τ*_0_ and *τ_m_*, respectively, while the slip values at these points are denoted by *s*_0_ and *s_m_*. The definitions of the strength (*τ_u_*) and slip (*s_u_*) at the peak slip point are provided in Section 3.2 and Section 3.3. Based on the test results presented in Table 5, the characteristic values mentioned above were statistically regressed. The calculation formulae for these characteristic values, which vary with the DS replacement ratio and the number of F-T cycles, are fitted and shown in Table 6.

The grouping of replacement ratios (R = 0, 0.4, 0.6 vs. R = 0.2, 0.8, 1.0) in Table 5 was based on distinct mechanical trends of τ values: under identical F-T cycles, specimens with R = 0, 0.4, and 0.6 consistently exhibited higher *τ* values, while those with R = 0.2, 0.8, and 1.0 showed significantly lower *τ* values.

### 4.2. Bond–Slip Model

**Figure 13:**
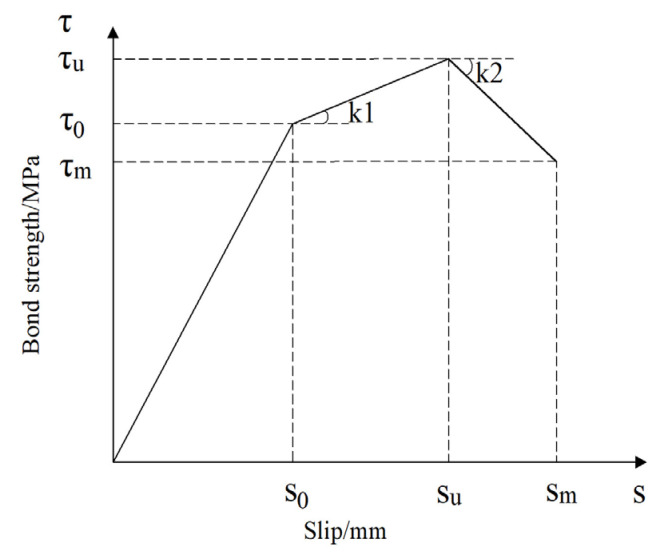
Bond–slip constitutive model.

As mentioned in Section 3.4, the second and third stages of the bond–slip curve exhibit nonlinear characteristics. For ease of application, these stages were simplified to linear relationships. Therefore, the bond–slip curve between DSC and steel bars after F-T cycles can be approximated using a three-stage linear model, as shown in Figure 13. This three-stage linear model is expressed by Equation (2). In this model, $k_{1}$ and $k_{2}$ represent the anchoring stiffness of each stage, i.e., the slope of the bond–slip curve. The values of *k_1_* and *k_2_* can be calculated using Equations (3) and (4), respectively. The values of *τ*_0_, *τ_u_*, and *τ_m_* for different specimens can be determined using the formulas in Table 6. Similarly, the values of *s*_0_, *s_u_*, and *s_m_* for different specimens can be taken from Table 5. By substituting *k_1_* and *k_2_* into Equation (2), the constitutive equations for pull-out specimens with different failure modes can be obtained. The bond–slip constitutive models for specimens corresponding to various failure modes are shown in Equations (5)–(6).

(2) $$\tau =\left\{\begin{array}{cc} \tau_{0} & \left(s=s_{0}\right)\\ \tau_{0}+k_{1}\left(s-s_{0}\right) & \left(s_{0}<s\leq s_{u}\right)\\ \tau_{u}-k_{2}\left(s-s_{u}-s_{0}\right) & \left(s_{u}<s\leq s_{m}\right)\\ \tau_{m} & \left(s=s_{m}\right) \end{array}\right.$$

(3) $$k_{1}=\frac{\tau_{u}-\tau_{0}}{s_{u}-s_{0}}$$

(4) $$k_{2}=\frac{\tau_{m}-\tau_{u}}{s_{m}-s_{u}-s_{0}}$$

(5) $$\tau =\left\{\begin{array}{cc} \tau_{0} & \left(s=s_{0}\right)\\ \tau_{0}+\frac{\tau_{u}-\tau_{0}}{s_{u}-s_{0}}\left(s-s_{0}\right) & \left(s_{0}<s\leq s_{u}\right)\\ \tau_{u} & \left(s=s_{u}\right) \end{array}\right.$$

(6) $$\tau =\left\{\begin{array}{cc} \tau_{0} & \left(s=s_{0}\right)\\ \tau_{0}+\frac{\tau_{u}-\tau_{0}}{s_{u}-s_{0}}\left(s-s_{0}\right) & \left(s_{0}<s\leq s_{u}\right)\\ \tau_{u}-\frac{\tau_{m}-\tau_{u}}{s_{m}-s_{u}-s_{0}}\left(s-s_{u}-s_{0}\right) & \left(s_{u}<s\leq s_{m}\right)\\ \tau_{m} & \left(s=s_{m}\right) \end{array}\right.$$

### 4.3. Validation of Bond–Slip Model

**Figure 14:**
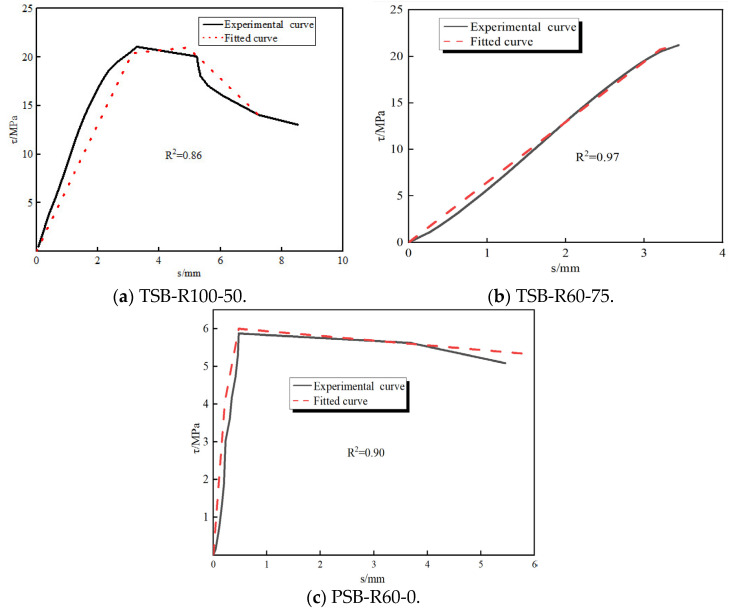
Comparison between fitted and experimental bond–slip curves.

The bond–slip model presented in Section 4.2 was used to fit the *τ*-*s* curves of PSB and TSB specimens. Figure 14 compares the fitted bond–slip curves with the experimental data, showing a strong agreement between the two. In conclusion, the bond–slip constitutive model proposed in this study effectively predicts the bond–slip behavior of steel bars in DSC after F-T cycles.

### 4.4. Comparison with Other Research Results

**Figure 15:**
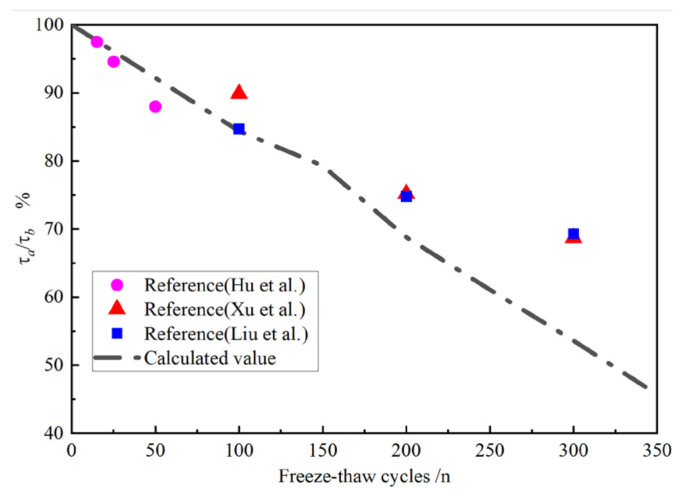
Comparison between the results of this study and those reported in the literature [42,43,44].

To verify the general applicability of the conclusions drawn in this study, relevant findings from other research [42,43,44] were selected for comparison. Figure 15 presents a comparison of the degradation in bond strength between ordinary concrete and steel bars at r = 0 under various numbers of F-T cycles (represented by the ratio of bond strength after F-T cycles to bond strength before F-T cycles, denoted as $\tau_{a}$/$\tau_{b}$). It can be observed that the bond strengths obtained in this study were close to those reported in previous studies. Moreover, the results were generally slightly conservative, indicating that they could be reliably used to predict the bond strength between steel bars and concrete subjected to F-T cycles.

## 5. Conclusions

A total of 144 specimens were tested to investigate the bond–slip performance of steel bars in DSC after F-T cycles, considering the effects of F-T cycles, DS replacement ratio, and reinforcement type. Following the experiments, the failure modes, bond–slip curves, and characteristic parameters of each specimen were obtained. Based on the experimental results, a simplified bond–slip constitutive model was proposed to describe the bond–slip behavior of steel bars in DSC, providing a valuable basis for practical engineering applications and design. The following conclusions can be drawn:

(1) After undergoing F-T cycles, all PSB specimens experienced pull-out failure. Meanwhile, most TSB specimens exhibited splitting failure, whereas a few specimens suffered a combined splitting and pull-out failure.
(2) Under identical conditions, the bond strength of PSB specimens was lower than that of TSB specimens. The bond strength between DSC and steel bars decreased with an increase in F-T cycles. For concrete incorporating 60% desert sand, compared to specimens without F-T cycles, the bond strength of PSB specimens decreased by 16.87% and 50.26% after 25 and 75 F-T cycles, respectively, while TSB specimens decreased by 2.33% and 9.5%, respectively.
(3) As the DS replacement ratio increased, the bond strength first decreased, then increased, and finally decreased again. When the F-T cycle was 0 times, compared with the sample with a DS replacement ratio of 0%, the bond strength of the PSB sample increased by −5.18%, 18.12%, 4.82%, −26.81%, and −31.25% at replacement rates of 20%, 40%, 60%, 80%, and 100%, respectively. The TSB samples increased by −3.22%, 5.98%, 1.42%, −1.59%, and −3.18%, respectively.
(4) Under identical conditions, the bond strength of PSB specimens was lower than that of TSB specimens. The bond strength between DSC and steel bars decreased with an increase in F-T cycles. As the DS replacement ratio increased, the bond strength first decreased, then increased, and finally decreased again.
(5) The bond–slip curve of the PSB specimen consisted of a linear rising stage, a slip-increase stage, and a debonding decline stage. In contrast, the bond–slip curve of the TSB specimen included a linear stage, a splitting failure stage, and a splitting–pulling stage.
(6) Based on statistical regression analysis of the experimental data, empirical formulae for calculating bond–slip characteristic values considering the influence of multiple factors were proposed. Furthermore, a bond–slip constitutive model for DSC and steel bars was established, which closely matched the experimental curves.
(7) The DSC samples with DS replacement ratios of 40% and 60% show that their bonding performance is significantly better than that of ordinary concrete samples, especially in cold environments. Therefore, it is recommended to use DSC samples with a substitution rate of 40%–60% to reduce the consumption of natural river sand in buildings in cold regions.

Although this study systematically investigated the bond behavior between DSC and steel reinforcement under F-T cycles, actual structures are exposed to multi-hazard environments involving coupled deterioration mechanisms such as F-T damage, chloride-induced corrosion, carbonation [45], and fire exposure [46]. Future work will focus on exploring pathways to enhance the bond performance of desert sand concrete with high replacement (≥80%) after F-T cycles through cement content optimization, superplasticizers, examining the evolutionary patterns of DSC-rebar bond performance under these complex interactions, with particular emphasis on fire-induced degradation mechanisms and post-fire residual capacity, employing beam specimen tests to systematically reveal structural levels. These investigations will establish multi-hazard durability design guidelines for DSC applications in cold regions and fire-prone infrastructures.

## Figures and Tables

**Table 1:** Value ranges of the main variables.

Variables	*R* (%)	*N*	Reinforcement Types
Values	0	0, 25, 50, 75	PSB
			TSB
	20	0, 25, 50, 75	PSB
			TSB
	40	0, 25, 50, 75	PSB
			TSB
	60	0, 25, 50, 75	PSB
			TSB
	80	0, 25, 50, 75	PSB
			TSB
	100	0, 25, 50, 75	PSB
			TSB

**Table 2:** Mix proportion and compressive strength of DSC.

R (%)	Mix Proportion (kg/m^3^)						Compressive Strength (MPa)
	Water	Cement	Water Reducer	Coarse Aggregate	River Sand	Desert Sand	
0	160	400	1.6	1288	552	0	36.10
20	160	400	1.6	1288	441.6	110.4	33.27
40	160	400	1.6	1288	331.2	220.8	39.70
60	160	400	1.6	1288	220.8	331.2	36.34
80	160	400	1.6	1288	110.4	441.6	34.63
100	160	400	1.6	1288	0	552	29.43

**Table 3:** Physical properties of aggregates.

Material	Bulk Density (kg/m^3^)	Apparent Density (kg/m^3^)	Water Absorption Rate (%)	water Content (%)	Mud Content (%)	Fineness Modulus (kg/m^3^)
crushed stone	1578	2840	0.8	0.5	0.9	-
Desert sand	1615	2630	2.1	1.5	1.9	0.198
River sand	1350	2038	0.8	1.9	2.2	2.58

**Table 4:** Mechanical properties of steel bars.

Types	Grades	d (mm)	Yield Strength (MPa)	Ultimate Strength (MPa)	Elastic Modulus (MPa)
TSB	HRB400	12	430	620	2 × 10^5^
PSB	HPB400	12	415	600	2 × 10^5^

**Table 5:** Test results of all pull-out specimens.

No.	*τ*_0_ (MPa)	*s*_0_ (mm)	*F_u_* (kN)	*s_u_* (mm)	SD of *s_u_*	*τ_u_* (MPa)	SD of *τ_u_*	*τ_m_* (MPa)	*s_m_* (mm)	Failure Mode
PSB-R0-0	3.78	0.36	12.67	0.53	0.04	5.60	0.16	5.52	2.22	P
PSB-R0-25	2.61	0.56	8.67	0.66	0.05	3.83	0.18	3.63	2.86	P
PSB-R0-50	2.46	0.35	7.75	0.87	0.05	3.42	0.19	3.13	2.68	P
PSB-R0-75	1.70	0.34	5.63	0.99	0.10	2.48	0.18	2.38	2.30	P
PSB-R20-0	3.41	0.44	12.02	0.68	0.06	5.31	0.19	4.82	7.24	P
PSB-R20-25	2.57	0.39	8.49	0.74	0.06	3.75	0.18	3.04	3.14	P
PSB-R20-50	2.37	0.22	7.35	0.96	0.09	3.25	0.17	1.90	1.87	P
PSB-R20-75	1.34	0.50	3.96	1.11	0.07	1.75	0.09	1.74	7.64	P
PSB-R40-0	4.36	0.35	15.95	0.5	0.03	7.05	0.40	6.52	1.33	P
PSB-R40-25	3.28	0.33	10.94	0.62	0.06	4.84	0.18	4.58	7.72	P
PSB-R40-50	2.59	0.26	8.57	0.75	0.06	3.79	0.31	2.63	5.92	P
PSB-R40-75	1.69	0.61	6.30	0.89	0.09	2.78	0.20	2.32	6.26	P
PSB-R60-0	4.17	0.34	13.28	0.48	0.05	5.87	0.31	5.08	5.45	P
PSB-R60-25	3.46	0.37	11.03	0.57	0.04	4.88	0.29	4.18	3.91	P
PSB-R60-50	2.13	0.42	7.71	0.73	0.05	3.41	0.21	1.98	4.52	P
PSB-R60-75	1.57	0.25	6.61	0.87	0.07	2.92	0.12	2.08	3.74	P
PSB-R80-0	3.52	0.47	9.18	0.59	0.05	4.06	0.27	4.04	1.19	P
PSB-R80-25	3.03	0.48	8.43	0.81	0.04	3.73	0.23	3.43	2.14	P
PSB-R80-50	1.47	0.16	6.60	1.02	0.08	2.92	0.19	2.32	3.30	P
PSB-R80-75	1.02	0.35	4.01	1.24	0.12	1.77	0.17	1.25	5.39	P
PSB-R100-0	3.04	0.42	8.70	0.61	0.05	3.85	0.18	2.99	4.30	P
PSB-R100-25	2.15	0.52	6.65	0.88	0.08	2.94	0.29	2.74	10.59	P
PSB-R100-50	1.68	0.56	4.41	1.15	0.09	1.95	0.14	1.57	6.64	P
PSB-R100-75	1.23	0.13	3.66	1.42	0.10	1.62	0.14	1.24	5.00	P
TSB-R0-0	22.55	2.60	52.57	2.97	0.29	23.24	1.35	-	-	S
TSB-R0-25	22.38	2.24	51.89	3.22	0.25	22.94	2.23	-	-	S
TSB-R0-50	21.46	2.28	49.50	3.45	0.34	21.88	1.38	-	-	S
TSB-R0-75	20.13	1.88	46.52	3.78	0.33	20.57	1.64	-	-	S
TSB-R20-0	21.71	1.40	50.86	3.10	0.16	22.49	1.62	-	-	S
TSB-R20-25	19.77	2.40	49.45	3.33	0.20	21.86	1.87	-	-	S
TSB-R20-50	19.94	2.07	48.35	3.62	0.32	21.38	1.38	-	-	S
TSB-R20-75	17.64	2.53	46.34	4.87	0.32	20.49	0.90	-	-	S
TSB-R40-0	23.78	2.26	55.71	2.42	0.19	24.63	1.22	-	-	S
TSB-R40-25	22.64	2.84	52.99	2.45	0.20	23.43	1.86	-	-	S
TSB-R40-50	21.82	2.58	50.94	2.79	0.14	22.52	1.88	-	-	S
TSB-R40-75	20.85	2.29	47.97	3.00	0.13	21.21	2.02	-	-	S
TSB-R60-0	19.80	1.90	53.32	2.67	0.08	23.57	1.69	-	-	S
TSB-R60-25	22.14	2.51	52.08	2.90	0.20	23.02	1.98	-	-	S
TSB-R60-50	21.64	2.68	49.74	3.13	0.11	21.99	1.63	-	-	S
TSB-R60-75	20.59	2.25	48.24	3.45	0.20	21.33	1.29	-	-	S
TSB-R80-0	20.91	2.45	51.73	3.87	0.27	22.87	1.21	-	-	S
TSB-R80-25	19.58	2.00	49.31	3.99	0.31	21.80	1.29	-	-	S
TSB-R80-50	19.32	2.01	48.66	4.16	0.17	21.51	1.05	-	-	S
TSB-R80-75	17.59	1.61	46.15	4.57	0.20	20.40	2.05	-	-	S
TSB-R100-0	20.11	1.82	50.89	2.99	0.19	22.50	1.34	-	-	S
TSB-R100-25	18.77	1.45	49.4	3.50	0.12	21.75	1.92	-	-	S
TSB-R100-50	18.56	2.33	47.67	3.91	0.15	21.07	1.59	14.03	7.25	S + P
TSB-R100-75	16.19	2.20	43.87	4.94	0.15	19.39	1.46	12.54	8.20	S + P

Note: *τ*_0_ denotes initial bond strength; *s*_0_ denotes initial slip; *F_u_* denotes peak load; *s_u_* denotes peak slip; SD of *s_u_* denotes standard deviation of peak slip; *τ_u_* denotes ultimate bond strength; SD of *τ_u_* denotes standard deviation of ultimate bond strength; *τ_m_* denotes debonding strength for PSB specimens and splitting pull-out strength for TSB specimens; *s_m_* denotes debonding slip for PSB and splitting slip for TSB.

**Table 6:** Calculation formulae for characteristic values of PSB and TSB specimens.

Steel Bar Types	Stages	Characteristic Values	Expressions
TSB	Linear	$\tau_{0}$	$\tau_{0}=\left\{\begin{array}{cc} \left(-0.085N+52.75\right)\left(0.019R+0.44\right) & R=0,\;0.4,\;\mathrm{and}\;0.6\\ \left(-0.112N+51.16\right)\left(-0.0311R+0.43\right) & R=0.2,\;0.8,\;\mathrm{and}\;1.0 \end{array}\right.$
	Splitting failure	$\tau_{u}$	$\tau_{u}=\left\{\begin{array}{cc} \left(-0.083N+53.48\right)\left(0.014R+0.443\right) & R=0,\;0.4,\;\mathrm{and}\;0.6\\ \left(-0.077N+51.21\right)\left(-0.005R+0.448\right) & R=0.2,\;0.8,\;\mathrm{and}\;1.0 \end{array}\right.$
	Splitting-pulling failure	$\tau_{m}$	$\tau_{m}=-0.0596N+17.01$
PSB	Linear rising	$\tau_{0}$	$\tau_{0}=\left\{\begin{array}{cc} \left(-0.205N+25.54\right)\left(0.0287R+0.148\right) & R=0,\;0.4,\;\mathrm{and}\;0.6\\ \left(-0.271N+31.54\right)\left(-0.017R+0.116\right) & R=0.2,\;0.8,\;\mathrm{and}\;1.0 \end{array}\right.$
	Slip increase	$\tau_{u}$	$\tau_{u}=\left\{\begin{array}{cc} \left(-0.233N+30.64\right)\left(0.041R+0.18\right) & R=0,\;0.4,\;\mathrm{and}\;0.6\\ \left(-0.247N+31.44\right)\left(-0.049R+0.17\right) & R=0.2,\;0.8,\;\mathrm{and}\;1.0 \end{array}\right.$
	Debonding decline	$\tau_{m}$	$\tau_{m}=\left\{\begin{array}{cc} \left(-0.269N+30.787\right)\left(0.003R+0.18\right) & R=0,\;0.4,\;\mathrm{and}\;0.6\\ \left(-0.311N+34.93\right)\left(-0.04R+0.134\right) & R=0.2,\;0.8,\;\mathrm{and}\;1.0 \end{array}\right.$

Note: Variable nomenclature is consistent with Table 5, unless otherwise specified.

## Data Availability

The original contributions presented in this study are included in the article. Further inquiries can be directed to the corresponding authors.
